# Effect of ionic strength on aggregation of nile red and coumarin 30 in aqueous medium: primary kinetic salt effect or salting-out effect?†

**DOI:** 10.1039/d3ra03829g

**Published:** 2023-08-23

**Authors:** Nitin Chattopadhyay, Arindam Das

**Affiliations:** a Department of Chemistry, Jadavpur University Kolkata 700 032 India nitin.chattopadhyay@yahoo.com

## Abstract

The effect of ionic strength on the aggregation of planar dyes like nile red (NR) and coumarin-30 (C30) in aqueous medium has been explored. The dyes are known to undergo dimerization, resulting in fading of their respective colors in the visible range. The present study demonstrates that the fading process is accelerated appreciably upon increasing ionic strength of the solution through addition of soluble salts. Experiments consist of variation of cations (Na^+^, Mg^2+^ and Al^3+^) with different valencies in a series of salts keeping the anion same and a similar set with a variation of anions (NO_3_^−^, SO_4_^2−^ and PO_4_^3−^), keeping the cation same. The question of involvement of ‘primary kinetic salt effect’ or ‘salting-out effect’ for accelerating the aggregation process has also been resolved. Using Na^+^, K^+^ and NH_4_^+^ ions with the same counterpart NO_3_^−^, our experimental results do not show any differential effect, in terms of making the aggregation process faster, and hence rule out any effect of Hofmeister series on the self-aggregation process. The detailed study explicitly establishes that it is the ‘primary kinetic salt effect’ and not the ‘salting-out effect’ that is involved in the present case.

## Abstract 2

Aggregation of nile red and coumarin 30 in aqueous medium is accelerated in presence of added salts. Simple absorption and emission based experiments assign primary kinetic salt effect, rather than salting-out effect, to be responsible for it.

## Graphical abstract

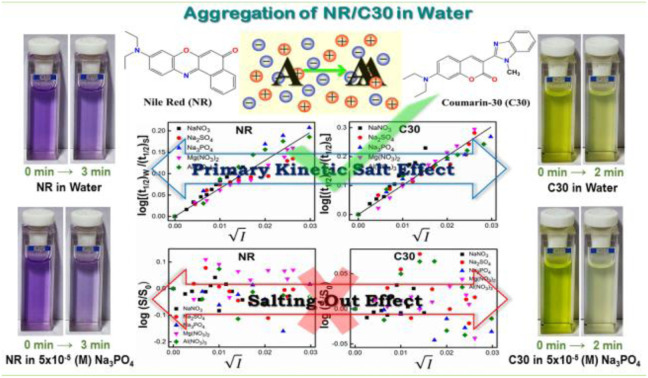

## Introduction

Fading of color of a planar dye in water due to aggregation is a well-known phenomenon. We have previously discussed the self-aggregation of dyes like coumarin-30 (C30), coumarin-7 (C7) and nile red (NR) in aqueous medium.^1,2^ In these reports, apart from pinpointing dimerization through non-covalent interactions to be the self-aggregation process and responsible for the observed color fading, we have further established that the self-aggregation process in aqueous medium can be controlled using cyclodextrins of proper dimension.^1,2^ Although considering its wide applications in diverse domains^3–6^ the importance of self-aggregation is now recognized, extension of the field addressing to its mechanistic aspect is still rare. Thus, whether or not the rate constant of the self-aggregation process depends on the ionic strength of aqueous salt solutions following the theories of Brønsted–Bjerrum and Debye–Hückel^7^ remains an open question requiring a thorough and explicit investigation. With this poor background, in the present work we intended to explore the effect of ionic strength on the aggregation process of the aforementioned dyes. It is evident from the experimental observations that with the addition of salts at low concentrations (where the Debye–Hückel limiting law stands) in aqueous solutions of the dyes, rate constant of the aggregation process increases significantly. This observation, however, invites two plausible and competing theories, namely, ‘primary kinetic salt effect’ and/or ‘salting-out effect’ for its explanation. Since none of the reacting species (NR and C30) are weak electrolytes, we did not consider a ‘secondary kinetic salt effect’. In this work we have successfully endeavored to resolve the issue, and that is the essence of our present investigation.

While the ‘primary kinetic salt effect’ is basically the effect of electrolyte concentration on the activity coefficients and thereby on the rate of a reaction, ‘salting-out’ is a phenomenon when the solubility of a nonelectrolyte substance in water decreases with increasing salt concentration in the solution.^8,9^ Contrary to the ‘salting-out’, in ‘salting-in’ the solubility of a nonelectrolyte in water increases with increasing salt concentration. The dye molecules under study being nonelectrolytes, logically one may invoke either of the effects depending on the experimental situation. Since the dye solutions are extremely dilute (in the range of 10^−5^ M) and we see only accelerated fading of the color of either of NR or C30 with the addition of dilute solutions of salts, we can easily rule out the possibility of ‘salting-in’. Nevertheless, we cannot rule out the possible involvement of ‘salting-out’ effect in a straight-forward manner. Anyway, since we are working only with solutions of low ionic strength, feasibility of it seems to be poor since ‘salting-out’ is significant only in a solution of high salt concentration (≥10^−1^ M). Through a series of experiments, the present report establishes that it is the ‘primary kinetic salt effect’ and not the ‘salting-out’ effect that is operative behind the faster fading of NR and C30 dyes in aqueous medium upon addition of dilute (∼10^−5^ M) solutions of salts.

**Scheme 1:**
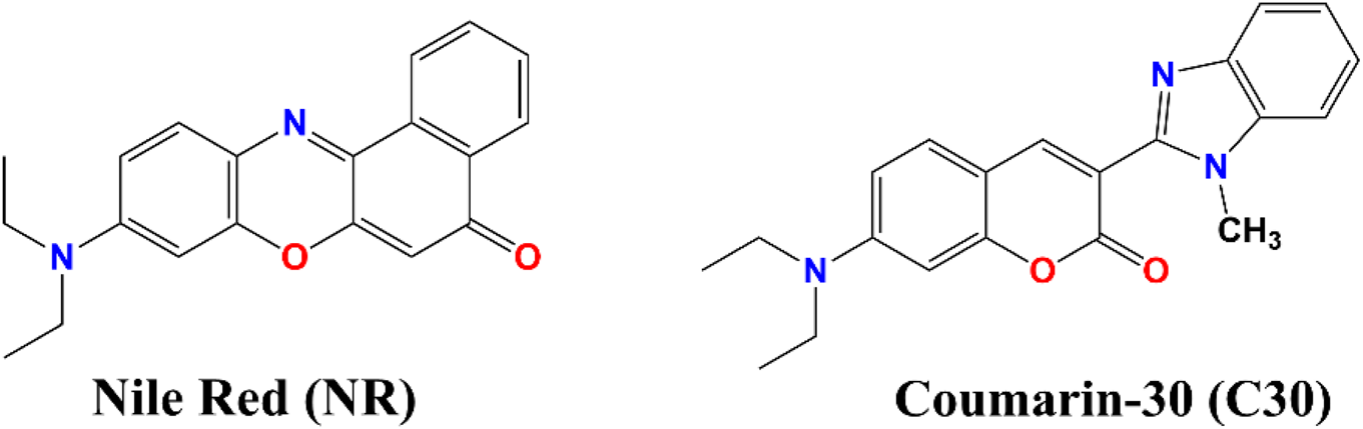
Structure of nile red (NR) and coumarin 30 (C30).

Effects of ionic activity are best understood in dilute solutions because coulombic interactions between ions dominate in dilute solutions. For this reason we have studied only in dilute solutions where salt concentrations are restricted in the range of 10^−5^ M. However, even at these low concentrations of added salts there is a possibility of a change of pH (considering salt hydrolysis, wherever applicable). Keeping this factor in mind, from our studied systems C7, C30 and NR, we have prudently chosen the latter two dyes (Scheme 1) as probes for the present study, leaving the first one (C7). Coumarin 7 (C7) possesses a dissociable hydrogen, which is sensitive to the pH of the medium, while for the chosen two probes this is not the case. Hence pH factor is not supposed to play a significant role in the present study. With the passage of time NR and C30 exhibit fading of color (reduction in their respective monomeric absorbance) signifying aggregation in aqueous medium, as reported before.^1,2^ From a preliminary experiment we also made an observation that with the addition of NaCl in solution, C7 and C30 dyes would prefer to form H-dimer to overcome the effect of ionic strength compared to the situation in pure water.^1^ Through the present work, we report a vivid pertaining study on the aspect of effect of ionic strength using several water soluble salts adopting simple absorption and fluorescence techniques. We have selected salts like NaNO_3_, Mg(NO_3_)_2_, Al(NO_3_)_3_, Na_2_SO_4_ and Na_3_PO_4_ to get two series where variation of cationic and anionic charge remains at +1, +2, +3 and −1, −2, −3 (monovalent → divalent → trivalent) respectively keeping the other ion same. Besides, we have also used another series, namely NaNO_3_, KNO_3_ and NH_4_NO_3_, where cation is varied keeping the charge (+1) and the anion (NO_3_^−^) same to check the role of Hofmeister series and therefrom the involvement of salting-out effect.

## Experimental section

### Materials

NR, C30 and all the anhydrous salts (purity min. 99.5%) used were purchased from TCI, India while spectroscopic grade methanol was purchased from Sigma-Aldrich (USA). All the chemicals were of good quality and were used without further purification. Milli-Q (Millipore) water was used throughout the experiment for performing the studies in aqueous medium.

### Methods

A Shimadzu UV-2450 absorption spectrophotometer (Shimadzu Corporation, Japan) was used to carry out absorption experiments. Fluorometric measurements were performed in a Horiba Jobin Yvon Fluoromax-4 spectrofluorometer.

Concentrated stock solutions of NR and C30 dyes were prepared separately in methanol as were done earlier.^1,2^ Small amount of stock solution of a dye was added to a cuvette with adequate amount of millipore water. It was then homogenised to perform the experiments. Volume of methanol in the final experimental solutions was kept below 2%.

For absorption and fluorometric measurements in the presence of salts, stock solutions of high concentrations (in the range of 10^−2^ M) of the desired salts were made. Using micropipette, calculated volumes of the salt solutions were added as per requirement directly to 3 mL of freshly prepared dye solutions taken in a fluorescence cuvette to make the salt concentrations in the range of 10^−5^ M. Absorption and fluorescence spectra were recorded as and when required. For the kinetic measurements absorbance or fluoresce intensity of the dye solution in the absence or presence of added salts were taken at corresponding *λ*_max_ for absorption and fluorescence spectra.

## Results and discussion

Although a text book topic, for the benefit of the general researchers and new entrants in this domain we would like to make a brief discussion on the basics relevant to the present study. Ionic strength of a solution (*I*) is a function of concentration of all the ions present in a solution, *i.e.*,1
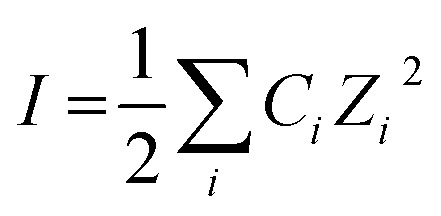
where *C*_*i*_ and *Z*_*i*_ are the molar concentration and the number of charge of the *i*th ionic component, respectively. Ionic strength of a solution, *i.e.*, the presence of ionic species in a solution, has an effect on the rate of a chemical reaction. This effect is known as kinetic salt effect. Primary kinetic salt effect is basically the influence of electrolyte concentration on the activity coefficients of the reactants and thereby on the rate of reaction.^10,11^ In aqueous medium the effect of ionic strength on the rate constant (*k*) of a second order reaction between ions is represented as^12^2
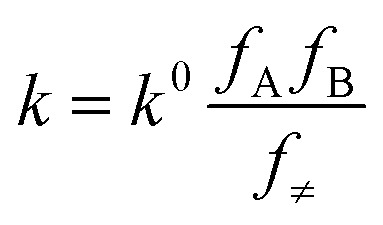
where *k*^0^ signifies the limiting value of the rate constant when the ionic strength tends to zero and *f*_A_, *f*_B_ and *f*_≠_ are activity coefficients of the reactant ions and the activated complex in transition state (TS) respectively. This is Brønsted equation (eqn (2)) that represents the influence of ionic concentration and charges (through activity coefficients, *f*) on the reaction rate in dilute solutions. Debye–Hückel limiting law (eqn (3)) provides the relationship between ionic strength (*I*) and activity coefficients (*f*) of the reacting ions in solution.3
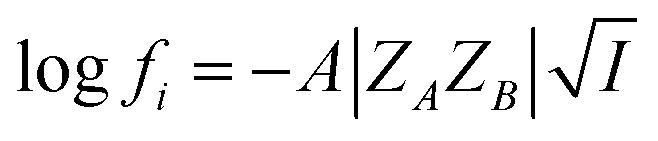
where *Z* is the ionic charge of the species denoted by the subscript and *A* is a constant that depends on the dielectric constant and temperature of the solution. By substituting eqn (3) in eqn (2), a relationship between the rate constant and ionic strength (*I*) is derived as^13^4
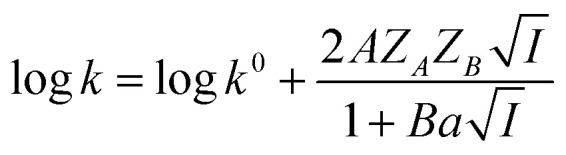
where *Z*_*A*_ and *Z*_*B*_ are the charges of the two reactants, *A* and *B* are constants, *A* = 0.509 and *B* = 0.33 (at *T* = 298 K, *ε*_Water_ = 80.3), *a* is the minimal distance (Å) between the two charges and *I* is in mol L^−1^. For reactions in dilute aqueous solutions, *i.e.*, at low ionic strength, the equation may be simplified as5
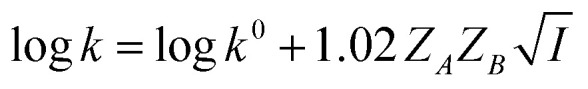


Eqn (5) is Brønsted–Bjerrum equation, represented in logarithmic form. Eqn (5) implies that a plot of 
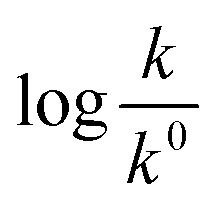
*versus*
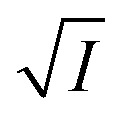
 should be linear passing through origin when primary kinetic salt effect is operative in a reacting system. The nature of slope is dictated by the product of charges on the species involved in the rate-limiting step. If the rate-limiting step involves species of like charges, a positive slope is expected. When the reaction is between opposite charges, it results in a negative slope.^14,15^ If one of the reactants is a neutral molecule (*Z*_*A*_*Z*_*B*_ = 0), the rate constant is expected to be independent of ionic strength.

On the other hand, the relative effectiveness of salting-out is usually calculated by the use of Setschenow equation^16^ (eqn (6)) in which *S*_0_ is the solubility of the solute in pure water, *S* is the solubility in the presence of salt, *K*_S_ is the salting-out or Setschenow constant, and *C* is the molar concentration of the salt. *K*_S_ will be positive when salting-out occurs and negative when salting-in occurs.^17,18^6
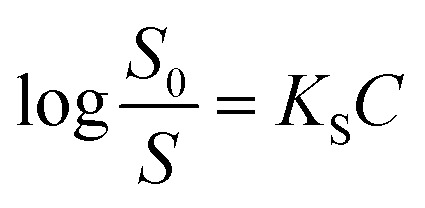


Using Debye–Hückel equation one can arrive at the following expression linking between solubility of a solute in aqueous medium and ionic strength of the solution in the presence of a salt at high ionic strength:^13,19,20^7
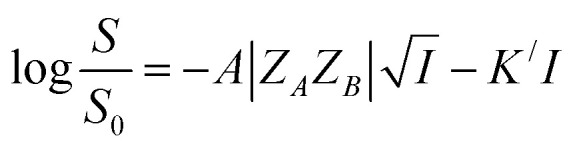
where *K*^/^ is a constant which depends on the size of solute and the ions present in the solution. Dividing eqn (7) by 
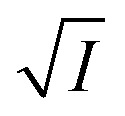
, we arrive at the following eqn (8);8
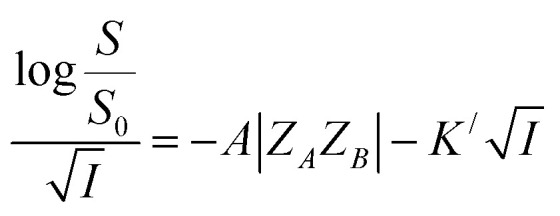


Eqn (8) implies that plot of 
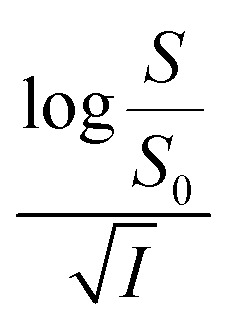
*versus*
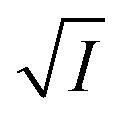
 should be linear with a negative slope *K*^/^ and an intercept −*A*|*Z*_*A*_*Z*_*B*_|.

We know that salting-out effect is observed at considerably high (≥10^−1^ M) salt concentrations.^11^ At higher salt concentrations, specific ion effects are also reported.^21–23^ The extent of specific ion effect depends on the following characteristics of the nonelectrolyte or the dye and it increases with (1) higher polarizability, (2) larger molecular size/volume and (3) lower polarity.^17^ The high valence electrical charges and small radii of ions are effective for salting-out according to Hofmeister series which is a foundational basis for selecting the correct salt for suitable salting-out effect.^24,25^ Generally high ionic charge in aqueous solution, because of its large Gibbs free energy of hydration, favor salting-out process.^26^ The reason behind salting-out is the combined effect of electronic repulsion and enhancement of the hydrophobic effect for dissolved cations or anions. We know that during nonelectrolyte solute/dye aggregation, increasing hydrophobic effect in pure water at the highly ordered solute–water interface diminishes the entropy. Likely, in the presence of salts of high charge density, similar surface associates are more ordered, suffering a large entropy reduction. This drop in entropy is overcome by the increase in entropy when large number of water molecules get free while de-solvating the solute. Therefore, highly water-solvated states of dyes are disfavored, instigating them to aggregate and then to leave the aqueous phase.^27,28^

### Absorption study

**Fig. 1:**
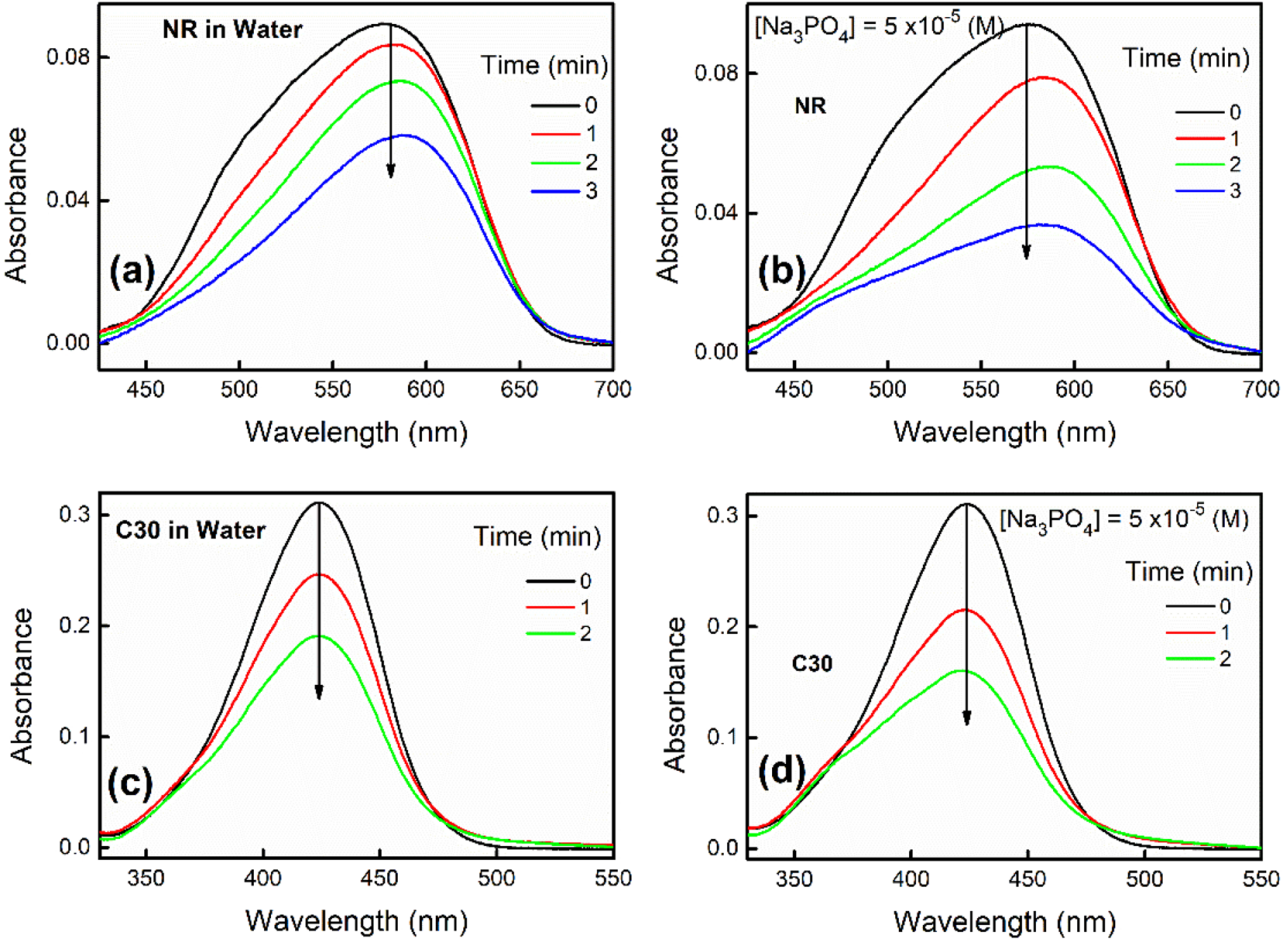
Absorption spectra of NR in (a) water and (b) 5 × 10^−5^ (M) Na_3_PO_4_ solution with increasing time. Absorption spectra of C30 in (c) water and (d) 5 × 10^−5^ (M) Na_3_PO_4_ solution with increasing time.

With passage of time NR and C30 display a decrease in their respective monomeric absorbance suggesting formation of H-dimer in aqueous medium as described in our previous contributions.^1,2^ Now, addition of a particular salt like Na_3_PO_4_ (10^−5^ M) results in a drastic enhancement in the rate of decrease in the absorbance as shown in Fig. 1.

We have also visibly observed the fading of color of NR and C30 in presence of different salts in water which are shown in Fig. S1–S4 in ESI.† To make a better perception, only selected sets (Fig. S1–S4†) are presented for color fading of aqueous solutions of NR (and C30) in the absence and presence of salts like Al(NO_3_)_3_ and Na_3_PO_4_, where the effect of ionic strength is found to be maximum, as expected. Thus, increase in ionic strength of solution is responsible for a faster decrease of absorbance or color fading of these dyes in aqueous medium.

### Fluorescence study

We have also looked at the fluorescence and fluorescence excitation spectra at different times to investigate self-association of these dyes in aqueous solutions and the effect of added salts on the rate of this process. Observation of a substantial decrease in the fluorescence intensities of NR and C30 with time in the presence of salt solutions compared to those in an aqueous medium (Fig. S5 in the ESI†), parallel to the absorption data, is attributed to self-aggregation of the dyes. Fluorescence excitation spectra of NR are resolved to get two very different spectra corresponding to the monomer and H-dimer species with *λ*_max_ 595 nm and 510 nm respectively. This is in gross agreement with the absorption results (being 580 nm and 475 nm respectively). Fluorescence excitation spectra of C30 in water are also resolved into two, resulting in *λ*_max_ 420 nm corresponding to the monomer and 370 nm for the dimer, in concurrence with the results obtained from resolution of the relevant absorption spectra (430 nm and 370 nm respectively). A set of such pictures for C30 are provided in Fig. S6 in the ESI,† as a sample, to demonstrate that the rate of aggregation process of the dye increases in the presence of salts. From these data it is clear that for both the dyes in aqueous solution with progress of time contribution of the monomer decreases and that of the dimer increases as observed earlier.^1,2^ At a fixed time, formation of the dimer follows the order: water < Na^+^ < Mg^2+^ < Al^3+^ and water < NO_3_^−^< SO_4_^2−^ < PO_4_^3−^ for salts with a common anion and a common cation respectively.

### Kinetic studies from absorption and fluorescence measurements

To study the kinetics of aggregation of NR and C30 in aqueous medium in the absence and presence of different salts from absorption measurements, absorbance values of the monomer of the dyes (at 580 nm for NR and 435 nm for C30) were monitored against time at a fixed dye concentration. A similar and parallel study was also performed monitoring the fluorescence intensities at the respective emission maxima of the dyes (660 nm and 500 nm for NR and C30 respectively) in the presence of different salts.

**Fig. 2:**
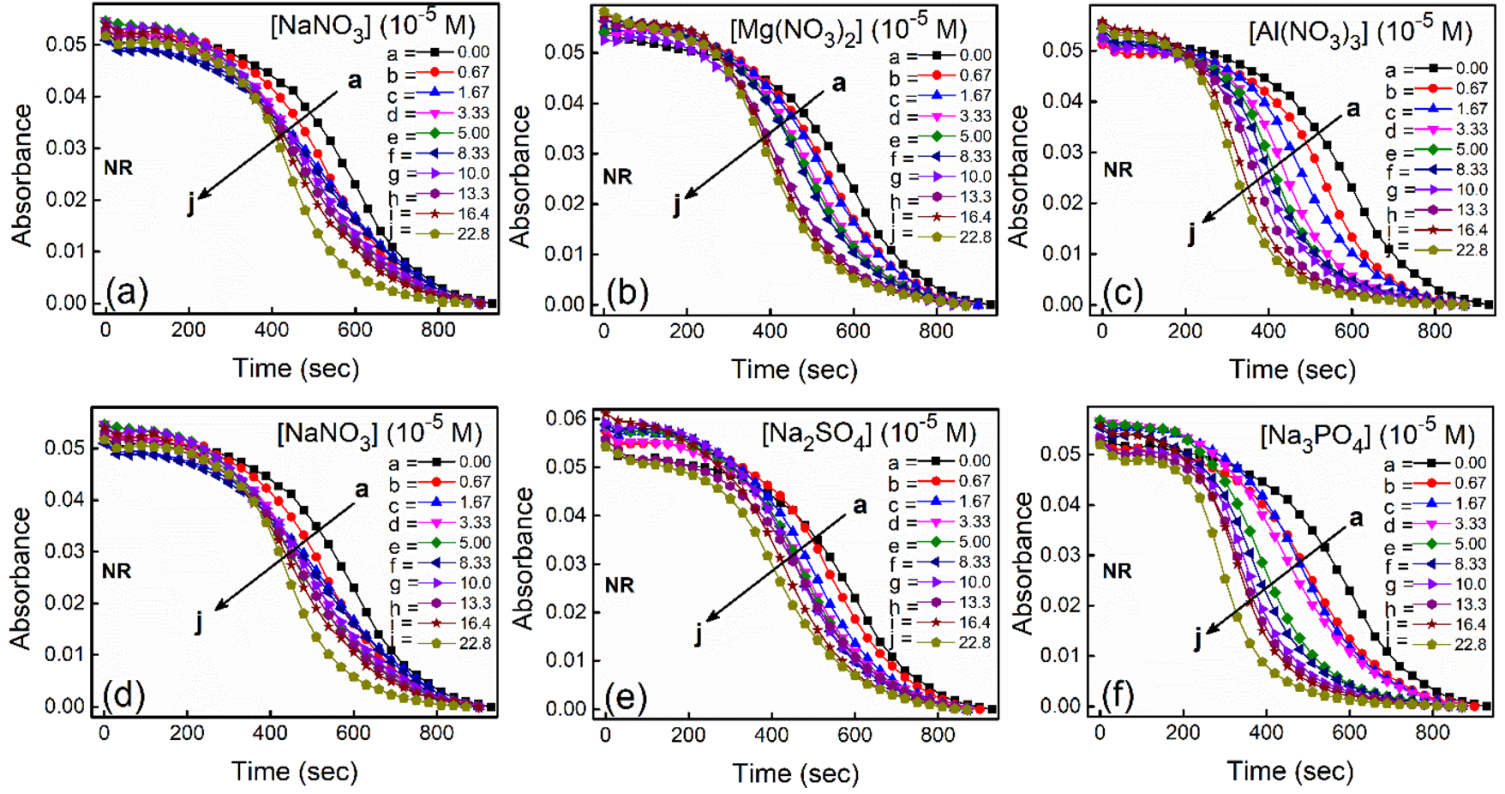
Absorbance kinetic decay of NR in water in presence of different salts with variable concentrations at *λ* = 580 nm. For a better visual comparison along upper or lower panel for the variation of cation and anion respectively, plots with NaNO_3_ are provided in both the panels.

**Fig. 3:**
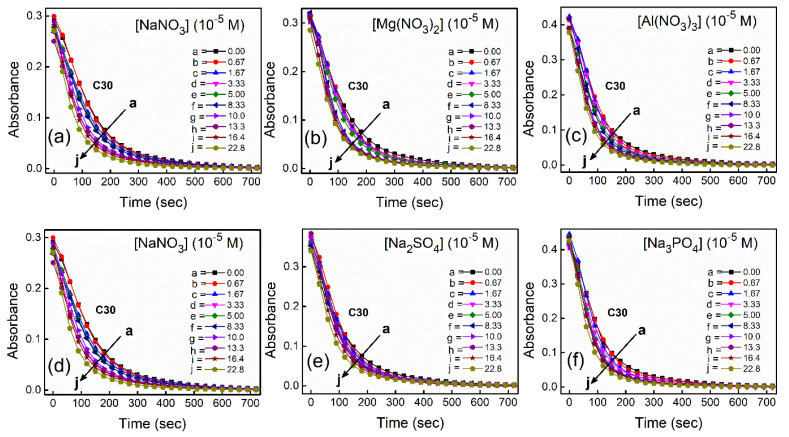
Absorbance kinetic decays of C30 in water in presence of different salts with variable concentrations at *λ* = 435 nm. For a better visual comparison along upper or lower panel for the variation of cation and anion respectively, plots with NaNO_3_ are provided in both the panels.

With increasing time, absorbance values of the dyes were found to decrease in water as well as in aqueous salt solutions. In the presence of salt solutions (∼10^−5^ M) the absorbance decays were faster compared to the decays in water for both the dyes. With increasing salt concentration the absorbance decays were gradually faster as shown in Fig. 2 for NR and in Fig. 3 for C30. As we moved from Na^+^ → Mg^2+^ → Al^3+^ the decays became progressively faster at a particular concentration of salt. Similarly, as we moved from NO^−^_3_ → SO^2−^_4_ → PO^3−^_4_ the decays were progressively faster. This observation was evident from the faster visible decolorization of these dyes in aqueous salt solutions as we moved from Na^+^ → Mg^2+^ → Al^3+^ and NO_3_^−^ → SO_4_^2−^ → PO_4_^3−^.

**Fig. 4:**
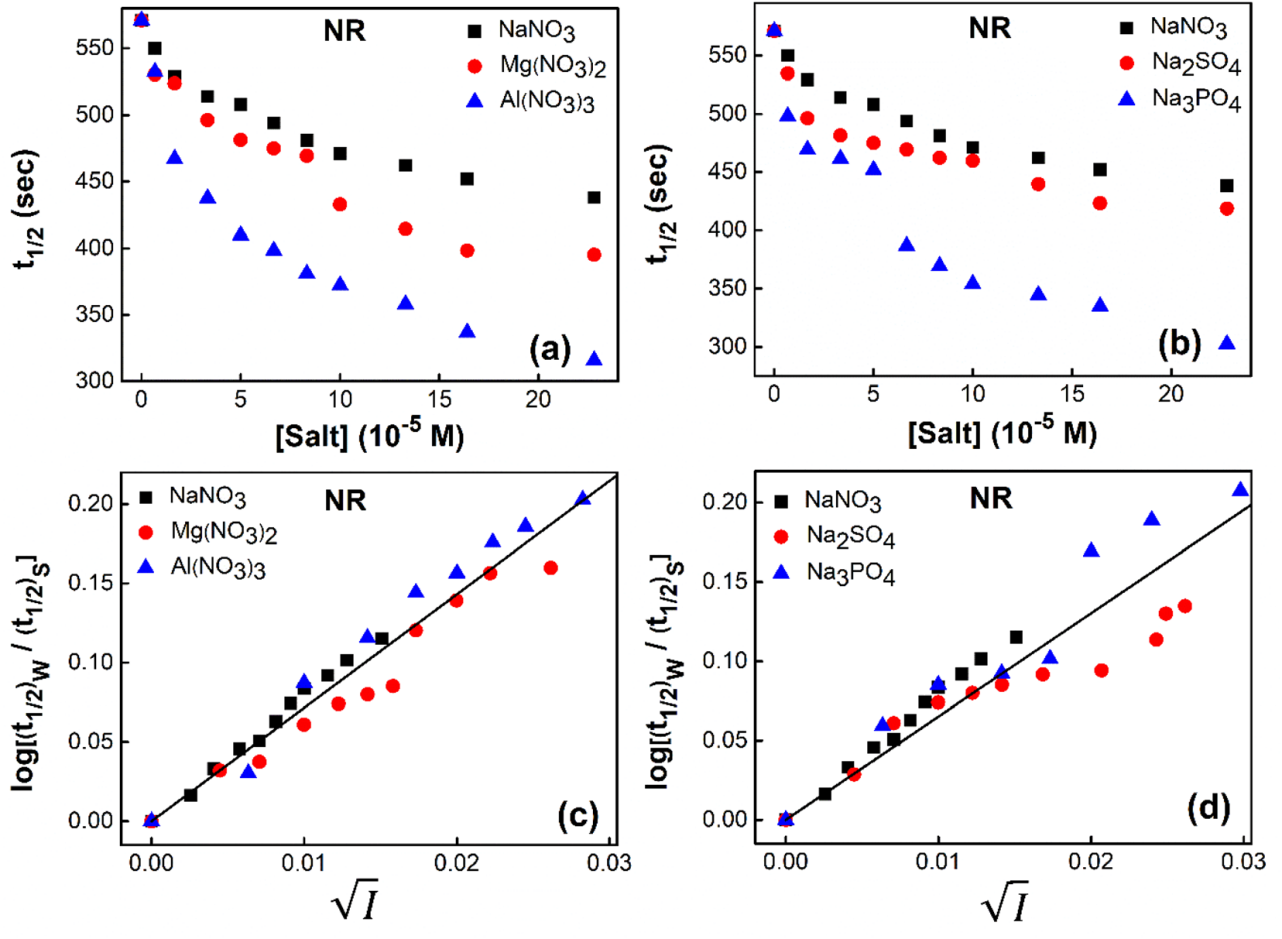
Variation of half-life (*t*_1/2_) *vs.* salt concentration for NR with a variation of (a) cation and (b) anion; (c) and (d) gives plots of log(*k*/*k*^0^) *vs.*
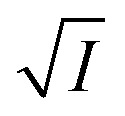
 for NR, where *k*/*k*^0^ = (*t*_1/2_)_W_/(*t*_1/2_)_S_.

Half-life (*t*_1/2_) values were determined from the decays of both the dyes in different solutions taking the absorbance values at the respective *λ*_max_. Since for a second order reaction *t*_1/2_ depends on the initial concentration of the reactant, for a relativistic study for our purpose, we determined *t*_1/2_ s keeping the dye concentration fixed for all the solutions. Hence at a fixed reactant (dye) concentration 
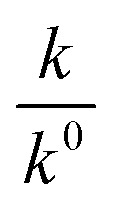
 turns out to be 
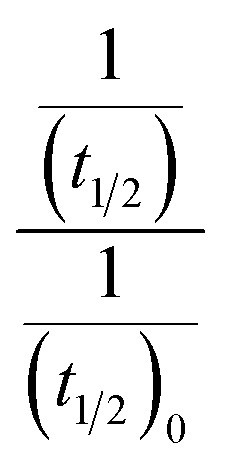
 , *i.e.*, 
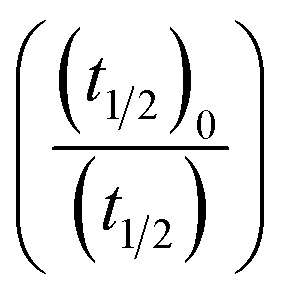
 ; where (*t*_1/2_)_0_ and (*t*_1/2_) denote the half-life of the dye molecule in solution in the absence and in the presence of salt respectively. Now eqn (5) becomes9
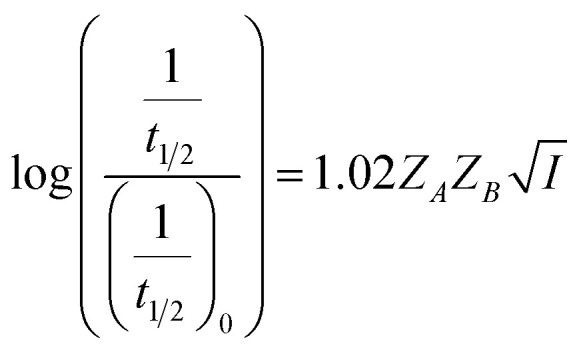
10
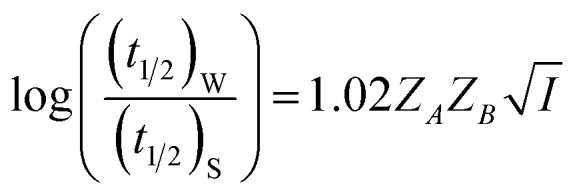
Finally (*t*_1/2_)_0_ and (*t*_1/2_) are replaced by more appropriate terms, (*t*_1/2_)_w_ and (*t*_1/2_)_S_, respectively for a better clarity referring the respective *t*_1/2_ s in water and salt solutions. The change in the half-life (*t*_1/2_)_S_ values of a fixed concentration of NR with increasing concentration of salts for cation variation (Na^+^ → Mg^2+^ → Al^3+^) and anion variation (NO_3_^−^ → SO_4_^2−^ → PO_4_^3−^) are shown in Fig. 4(a) and (b) respectively. Similar plots of half-life (*t*_1/2_)_*S*_ at a fixed concentration of C30 *versus* salt concentration for cation variation (Na^+^ → Mg^2+^ → Al^3+^) and anion variation (NO_3_^−^ → SO_4_^2−^ → PO_4_^3−^) are shown in Fig. S7(a) and (b) respectively in the ESI.† The plots reveal that half-life (*t*_1/2_)_*S*_ of both the dyes decrease at a faster rate as the charge of cation (Na^+^ < Mg^2+^ < Al^3+^) or anion (NO_3_^−^ < SO_4_^2−^ < PO_4_^3−^) increases, keeping the counter ion same.

**Fig. 5:**
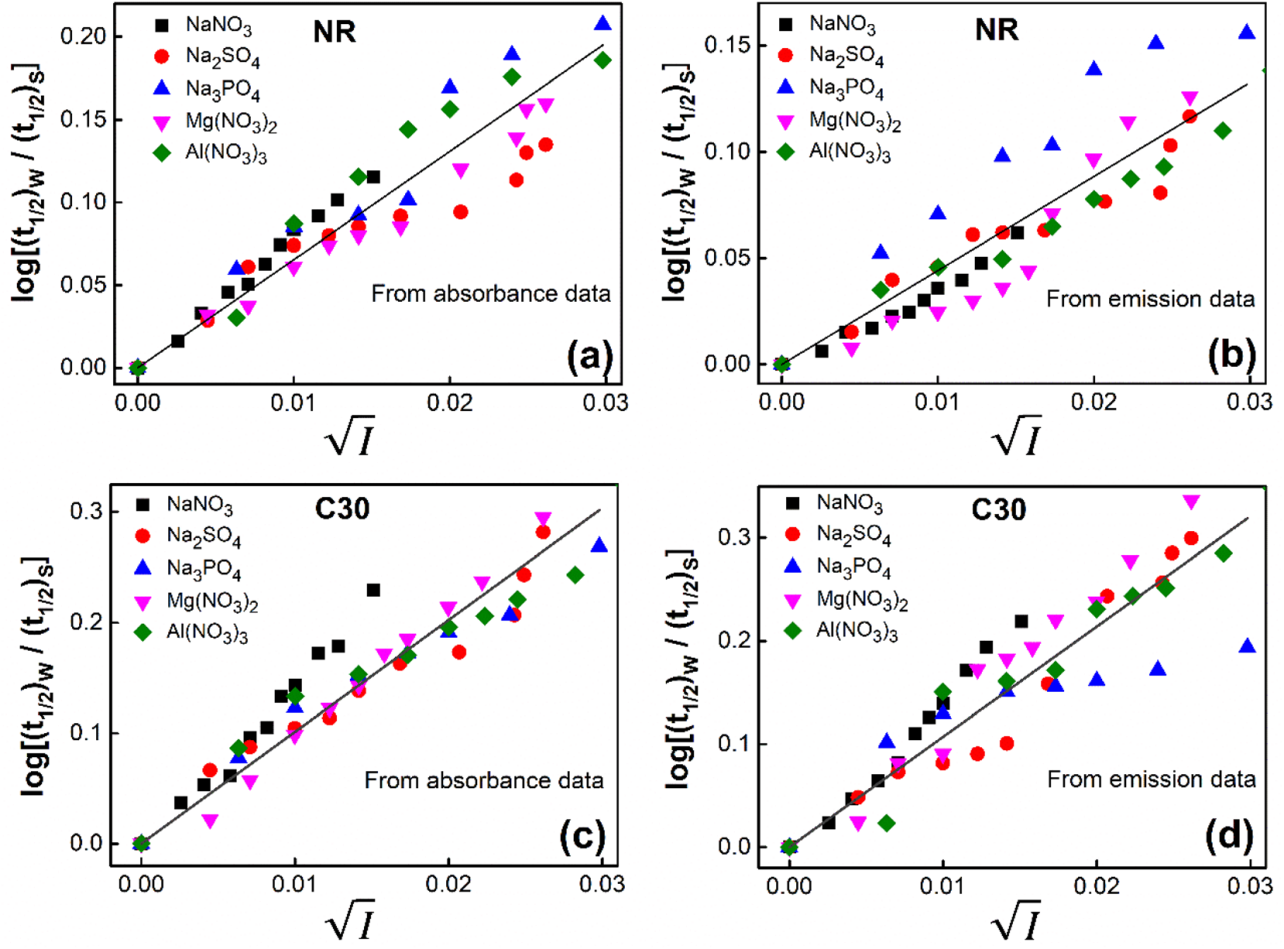
Plot of log(*k*/*k*^0^) *vs.*
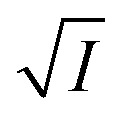
 of NR in water with variation of salts from (a) absorbance data and (b) emission data, where *k*/*k*^0^=(*t*_1/2_)_w_/(*t*_1/2_)_S_. (c) and (d) represent similar plots for C30.

Now, plots of 
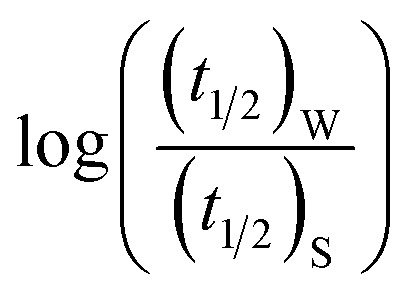
*versus*
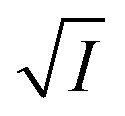
 (eqn (10)) for NR are presented in Fig. 4(c) and (d); former corresponding to cation variation (Na^+^ → Mg^2+^ → Al^3+^) while the latter to anion variation (NO_3_^−^ → SO_4_^2−^ → PO_4_^3−^). Although scattered, the plots are found to follow linearity (passing through origin) implying that Brønsted–Bjerrum equation (eqn (5)) is obeyed for both the series of salts. Similar plots for C30 are depicted in Fig. S7(c) and (d) in the ESI.† For a wider perception we have plotted 
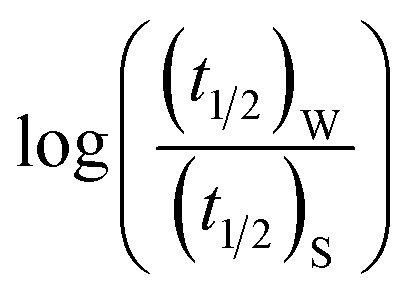
*versus*
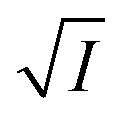
 for NR and C30 with all the five salts NaNO_3_, Mg(NO_3_)_2_, Al(NO_3_)_3_, Na_2_SO_4_ and Na_3_PO_4_ together revealing that the entire data set follows linearity passing through the origin (Fig. 5(a) and (c) respectively).

To validate reliability of the absorption based kinetic data, fluorescence decays of the probes were also followed. The results of the fluorometric study are presented pictorially in Fig. S8 and S9 in the ESI† for NR and C30 respectively. 
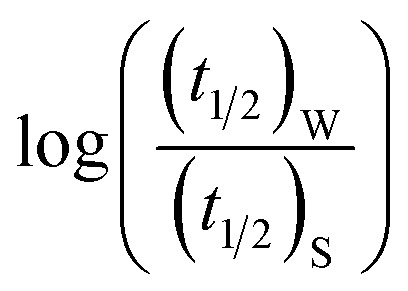
*vs.*
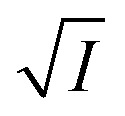
 plots have been made independently from fluorescence kinetic data following the similar procedure as adopted with absorption based kinetic study. These are presented in Fig. 5(b) and (d), together with the corresponding plots developed from the absorption kinetic data. It is evident from these figures that within experimental limits the absorption and fluorescence kinetic data sets corroborate one another.

Thus, all our experimental results, based on absorption and fluorescence kinetic data, conform to Brønsted–Bjerrum theory (eqn (5)) and thereby substantiate the involvement of primary kinetic salt effect in the present study ratifying a faster rate of aggregation of NR and C30 with an increase in salt concentration in the dye solutions in aqueous medium.

Since we were restricted to addition of only very dilute solutions of salts (∼10^−5^ M) to the dye solutions, involvement of salting-out effect seems unrealistic. However to eliminate its possible involvement completely, we have followed two approaches.

**Fig. 6:**
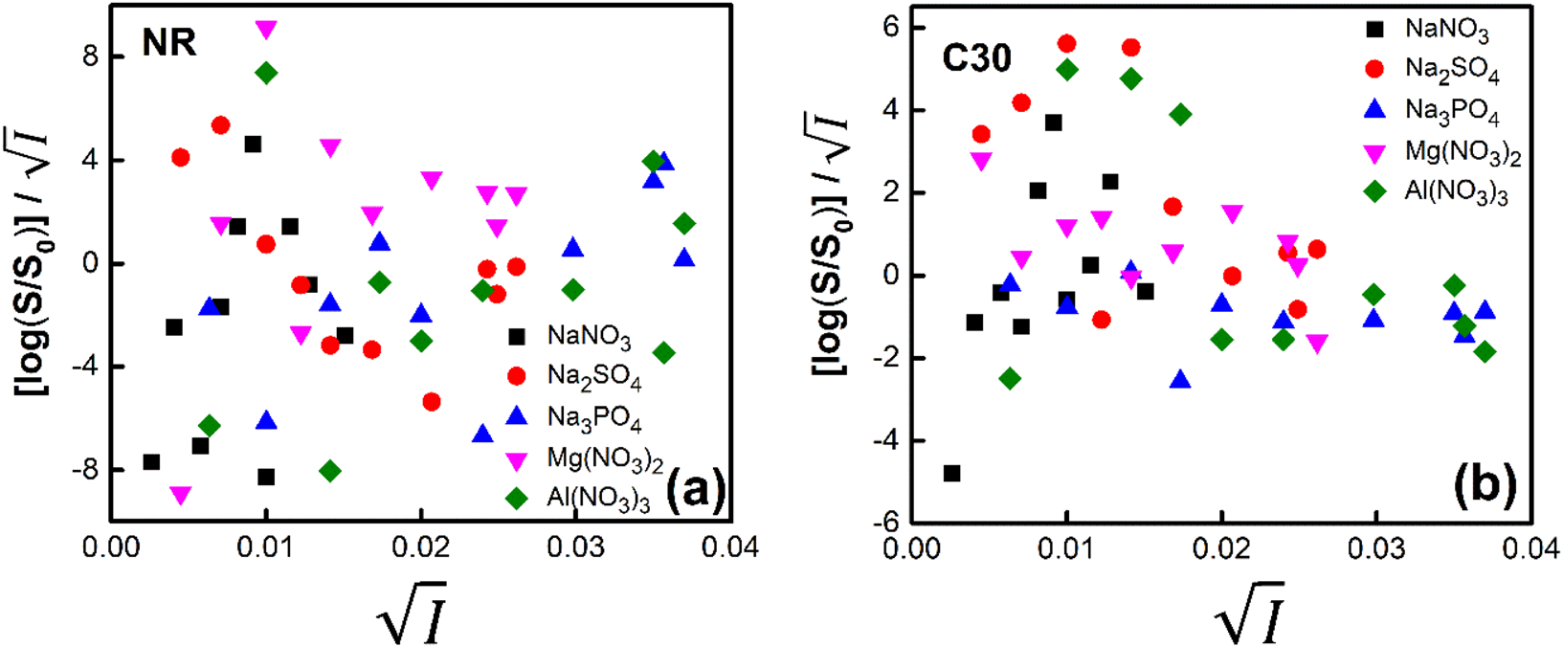
Plot of 
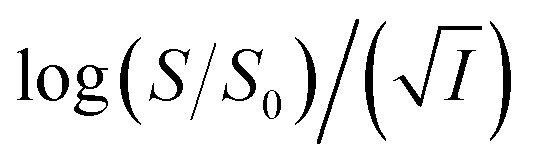
*vs.*
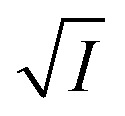
 for (a) NR and (b) C30 in water with variation of salts from absorption measurements.

Firstly, we intended to plot 
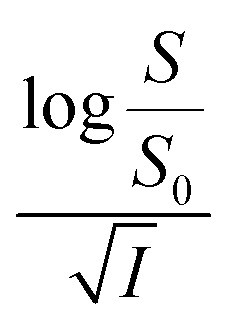
*versus*
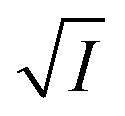
 to see whether eqn (8), relevant for the salting-out effect, is followed or not. For the purpose, we have taken the following strategy. Accepting validity of Beer's law, meaning concentration of a soluble entity is proportional to its absorbance (optical path length being the same, here 1 cm), we have taken ratio of the absorbance value of a fixed concentration of a dye (monomer) in water to the same in the presence of a known concentration of the added salt as *S*_0_/*S*, ratio of the respective concentrations. Both the absorbance values were taken after attainment of equilibrium (for both NR and C30, 15.0 minutes after making the solution), when absorbance attained a steady value in each case. To monitor the change in absorbance of the monomer species of an individual dye in the absence and presence of the added salt, we chose wavelengths corresponding to the maximum (*λ*_max_) in the respective absorption spectrum, namely 580 nm and 435 nm for NR and C30 respectively. Then 
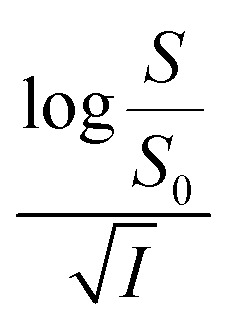
 was plotted against 
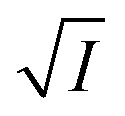
 for all individual salts added (Fig. 6). The figure depicts no definite pattern followed by the different salts, revealing clearly that eqn (8) is not obeyed for the present systems. This experiment, therefore, rules out involvement of salting-out effect for the cases studied.

**Fig. 7:**
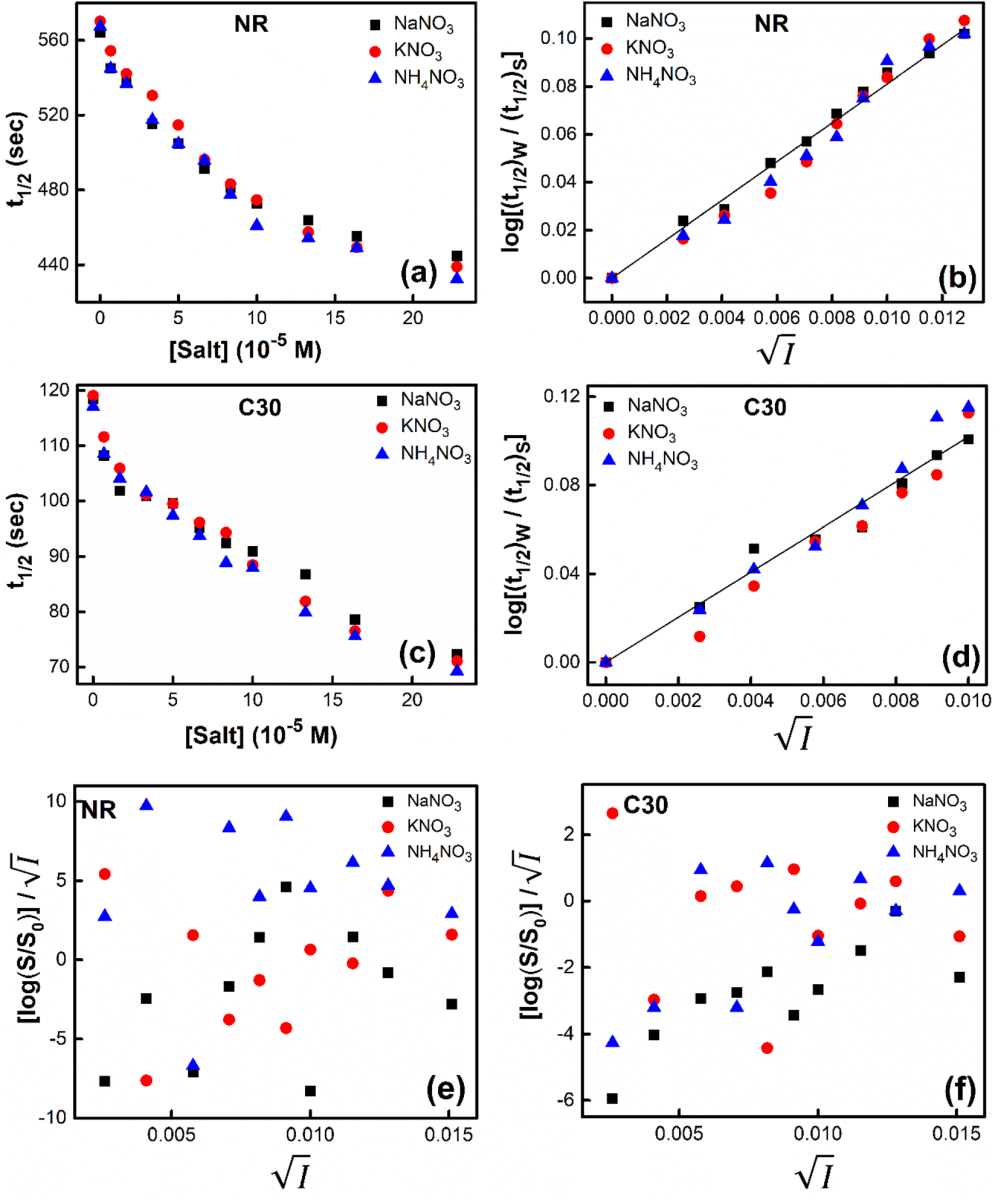
Plot of half-life (*t*_1/2_) *vs.* salt concentration for (a) NR and (c) C30 with variation of concentration of Na^+^, K^+^ and NH_4_^+^ ions. Plot of log(*k*/*k*^0^) *vs.*
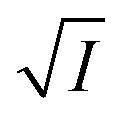
 for (b) NR and (d) C30, where *k*/*k*^0^=(*t*_1/2_)_w_/(*t*_1/2_)_S_. Plots of 
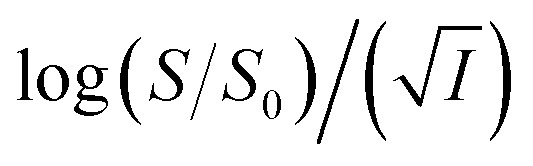
*vs.*
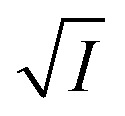
 for (e) NR and (f) C30 in water with variation of concentrations of NaNO_3_, KNO_3_ and NH_4_NO_3_ salts, from absorption based measurements.

Secondly, we tried to check the validity of Hofmeister series,^24^ which is a signature of the involvement of salting-out effect in the process under consideration. For the purpose, we chose monovalent cations like Na^+^, K^+^ and NH^+^_4_ with the monovalent and same anion (NO_3_^−^) using NaNO_3_, KNO_3_ and NH_4_NO_3_ salts. We studied the problem from two angles. From kinetic point of view, variation of *t*_1/2_ with a change of concentration of these salts added individually in aqueous solutions of NR and C30 respectively were noted (Fig. 7).

The figure reveals that at a particular concentration all these salts (both the constituent ions being monovalent) affect the aggregation process to the same extent. It was further clarified from the plot of 
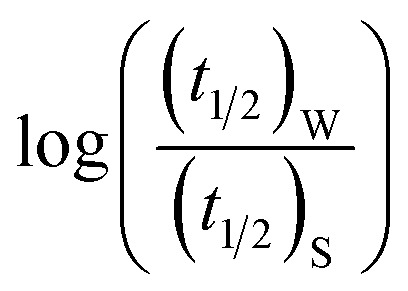
 against 
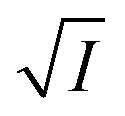
 taking the absorption kinetic data of the two dyes with the added salts at various concentrations (Fig. 7). The figure reveals that the experimental data points with all the three salts could be fitted on a single straight line passing through the origin revealing that there is hardly any differential effect of Na^+^, K^+^ and NH^+^_4_ ions in terms of accelerating the color fading of the dyes. By the way, an apparent biexponential nature observed in Fig. 7(c) is presumably associated with the inherent experimental error. There is no logic for the pattern observed in Fig. 7(c) to be different from that in Fig. 7(a). This, of course, does not affect anyway the overall outcome of this experiment as evident from Fig. 7(b) and (d). Following the strategy as stated in the previous paragraph, we have also plotted 
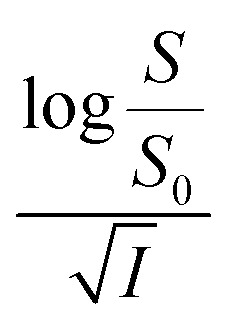
 against 
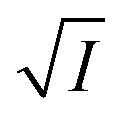
 for gradual addition of these three salts (Fig. 7(e) and (f)). Here also we could not notice any differential effect nor there was a pattern of the data points as one expects from eqn (8), had there been salting-out effect operative. Both these experiments raise question on the applicability of Hofmeister series for the present problem of self-aggregation of NR and C30. Involvement of salting-out effect was therefore ruled out for the present systems leaving no room other than to accept the exclusive involvement of primary kinetic salt effect to accelerate the aggregation process of the two studied dyes leading to the color fading in the presence of added salts.

Thus, all our experimental results portray primary kinetic salt effect to be responsible for the faster color fading of aqueous solutions of the studied dyes (NR and C30) in the presence of salts and eliminate involvement of salting-out effect during the process. It is pertinent to mention here that the present study opens up an interesting issue to think on. Apparently, for the present cases both the molecules of NR (or C30), taking part in the dimerization reaction, are uncharged; *i.e.*, *Z*_*A*_ and *Z*_*B*_ are both zero. As has been discussed above, for non-ionic reactants slope of a plot corresponding to eqn (5) is expected to be zero (*Z*_*A*_ and/or *Z*_*B*_ being 0). In contrast, our absorption and emission based experimental results (Fig. 5) reveal clear positive slopes, indicating that somehow there is development of partial charges on the reacting species during reaction. Although our reactants (monomers of NR or C30) appear non-ionic, they are dipolar in nature. Keeping in mind self-aggregation of the studied dyes (monomer → dimer) as the reaction, we feel that this dipolar nature of the individual dyes plays a crucial role in the process. Further, from a qualitative point of view, a higher slope of plots of 
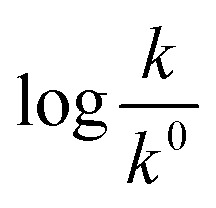
*vs.*
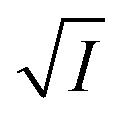
 in case of C30 as compared to that for NR (evident from a comparative glance on Fig. 5, *i.e.*, ratio of slopes for C30 and NR is 1 : 0.7 from absorption measurements, 1 : 0.45 from emission measurements and 1 : 0.57 from the average of slopes obtained from the two measurements) gets a qualitative support from a higher value of the calculated dipole moment of C30 (12.1807 D) as to that of NR (9.9851 D) obtained from the respective optimized structures. A more detail study on various molecular systems is, however, invited in this line to really resolve the issue.

## Conclusions

Effect of ionic strength of solution on the self-aggregation (dimerization) process of two dyes, NR and C30, leading to color fading of their aqueous solutions, has been studied in detail through simple absorption and fluorescence based measurements. For the purpose, dilute solutions of various salts were used to make series like Na^+^, Mg^2+^ and Al^3+^ as well as NO_3_^−^, SO_4_^2−^ and PO_4_^3−^ (in the former case character of the cation and in the latter case that of the anion varies keeping the counter ion same in each series). The detailed study revealed that Brønsted–Bjerrum equation is obeyed by salts of both the series signifying primary kinetic salt effect to be operative. Experimental data points with all the different salts could be fitted on the same straight line. On the other hand, possible involvement of salting-out effect was ruled out from absorption and fluorescence based data as well as from the absence of any differential effect of Na^+^, K^+^ and NH_4_^+^ on the process, implying that Hofmeister series, which is a signature of salting-out effect, is not operative during the aggregation process of these dye systems. All the experimental observations portray exclusive involvement of primary kinetic salt effect on the self-aggregation process of both the studied dyes ruling out any role of salting-out effect.

## Author contributions

NC conceptualized and supervised the project and analyzed the experimental data. AD performed all the experiments.

## Conflicts of interest

The authors declare no conflict of interest.

## Supplementary Material

RA-013-D3RA03829G-s001

## References

[cit1] Das A., Das S., Biswas A., Chattopadhyay N. (2021). J. Phys. Chem. B.

[cit2] Ray A., Das S., Chattopadhyay N. (2019). ACS Omega.

[cit3] Sengupta S., Würthner F. (2013). Acc. Chem. Res..

[cit4] Li K., Duan X., Jiang Z., Ding D., Chen Y., Zhang G. -Q., Liu Z. (2021). Nat. Commun..

[cit5] Verma P., Pal H. (2014). J. Phys. Chem. A.

[cit6] Xu F., Testoff T. T., Wang L., Zhou X. (2020). Molecules.

[cit7] Fedoseeva M., Fita P., Punzi A., Vauthey E. (2010). J. Phys. Chem. C.

[cit8] Randall M., Failey C. F. (1927). Chem. Rev..

[cit9] Gross P. M. (1933). Chem. Rev..

[cit10] Zhang C., Qu Y., Zhao X., Zhou Q. (2015). Clean: Soil, Air, Water.

[cit11] Atkins P. , Atkins P. W. and de Paula J. C., Atkins' Physical Chemistry, Oxford University Press, 2014

[cit12] Logan S. (1967). Trans. Faraday Soc..

[cit13] Hofer P., Fringeli U. P., Hopff W. H. (1984). Biochem.

[cit14] Jonnalagadda S., Gollapalli N. (2000). J. Chem. Educ..

[cit15] Alibrandi G., Coppolino S., D'Aliberti S., Ficarra P., Micali N., Villari A. (2003). J. Pharm. Sci..

[cit16] Setschenow J. (1889). Z. Phys. Chem..

[cit17] Hyde A. M., Zultanski S. L., Waldman J. H., Zhong Y.-L., Shevlin M., Peng F. (2017). Org. Process Res. Dev..

[cit18] Sergeeva V. (1965). Russ. Chem. Rev..

[cit19] Brunet M. , OER by Discipline Guide, University of Ottawa Library, 2010, v. 1.0–June 2021

[cit20] Dennis C. R., Potgieter I., Basson S. (2010). React. Kinet., Mech. Catal..

[cit21] Leberman R. (1991). FEBS Lett..

[cit22] Tobias D. J., Hemminger J. C. (2008). Science.

[cit23] Görgényi M., Dewulf J., Van Langenhove H., Héberger K. (2006). Chemosphere.

[cit24] Hofmeister F. (1888). Arch. Exp. Path. Pharm..

[cit25] Kunz W., Henle J., Ninham B. W. (2004). Curr. Opin. Colloid Interface Sci..

[cit26] Wu H. S., Wang Y. J. (2012). Ind. Eng. Chem. Res..

[cit27] Grover P. K., Ryall R. L. (2005). Chem. Rev..

[cit28] Graziano G. (2010). Chem. Phys. Lett..

