# Supplementary material for: Effect of ionic strength on aggregation of nile red and coumarin 30 in aqueous medium: primary kinetic salt effect or salting-out effect?
Source: RSC Adv. 2023 Aug 23;13(36):25159–68. doi: 10.1039/d3ra03829g (PMC10445056; doi:10.1039/d3ra03829g)
Supplement: RA-013-D3RA03829G-s001 [file RA-013-D3RA03829G-s001.pdf]

Electronic Supplementary Material (ESI) for RSC Advances.
This journal is © The Royal Society of Chemistry 2023

Effect of Ionic Strength on Aggregation of Nile Red and Coumarin 30 in Aqueous Medium: Primary Kinetic Salt Effect or Salting-out Effect?

Nitin Chattopadhyay*, Arindam Das
Department of Chemistry, Jadavpur University, Kolkata - 700 032, India
*Corresponding author.
E-mail: nitin.chattopadhyay@yahoo.com

Electronic Supplementary Information (ESI)

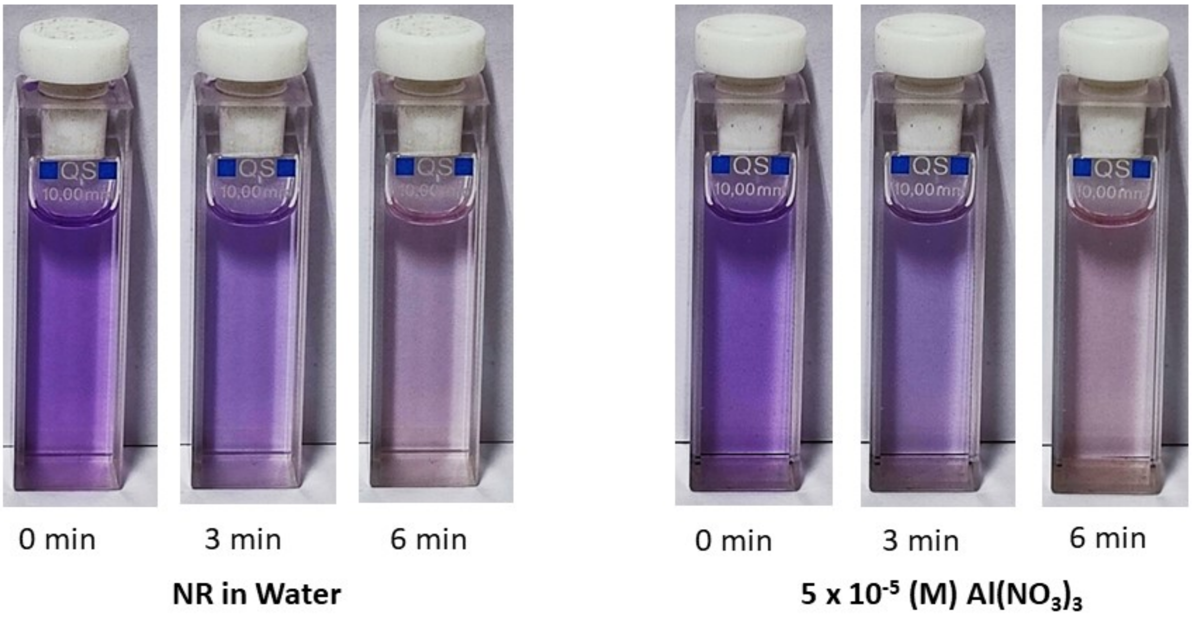

Figure S1: Snap shots of NR in water and in 5 × 10^{-5} (M) $Al(NO_3)_3$ solution at different times.

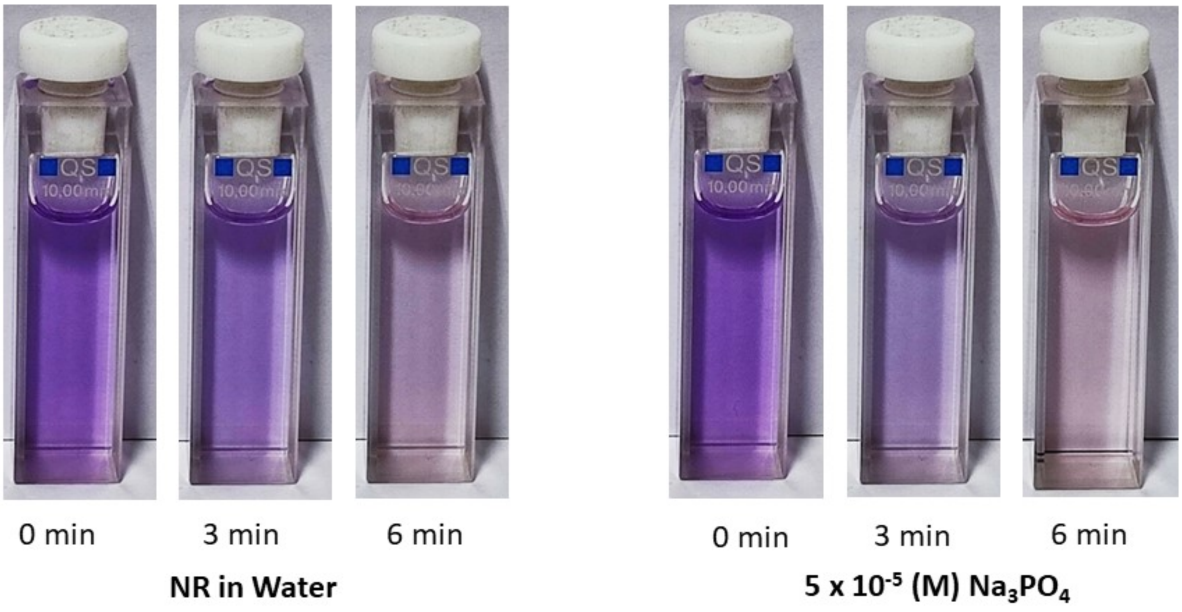

Figure S2: Snap shots of NR in water and in 5 × 10^{-5} (M) Na_3PO_4 solution at different times.

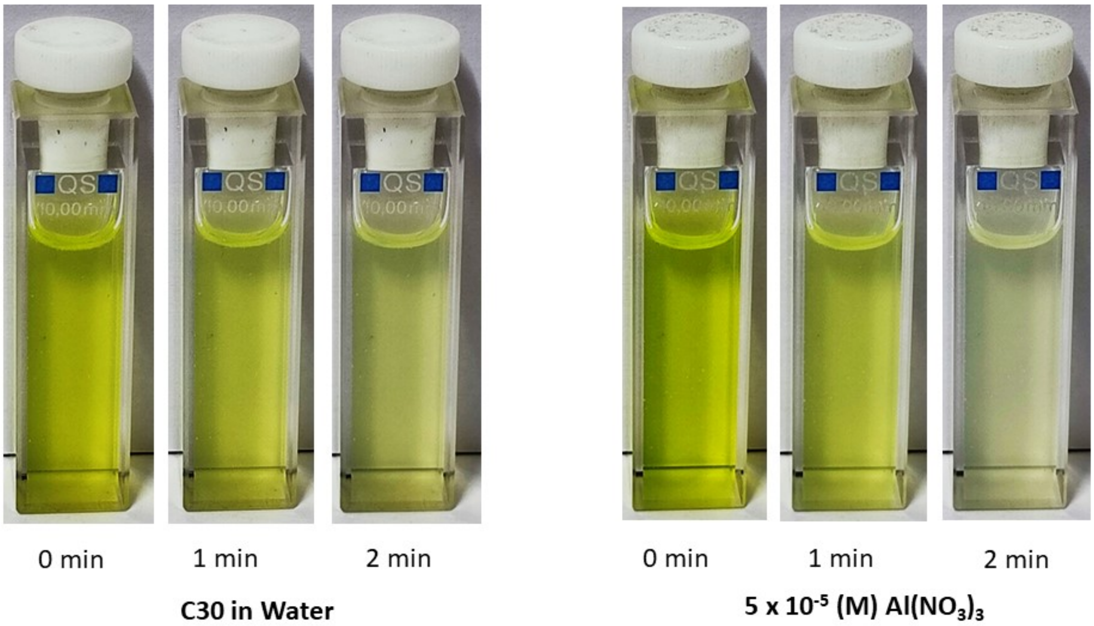

Figure S3: Snap shots of C30 in water and in 5 × 10^{-5} (M) $Al(NO_3)_3$ solution at different times.

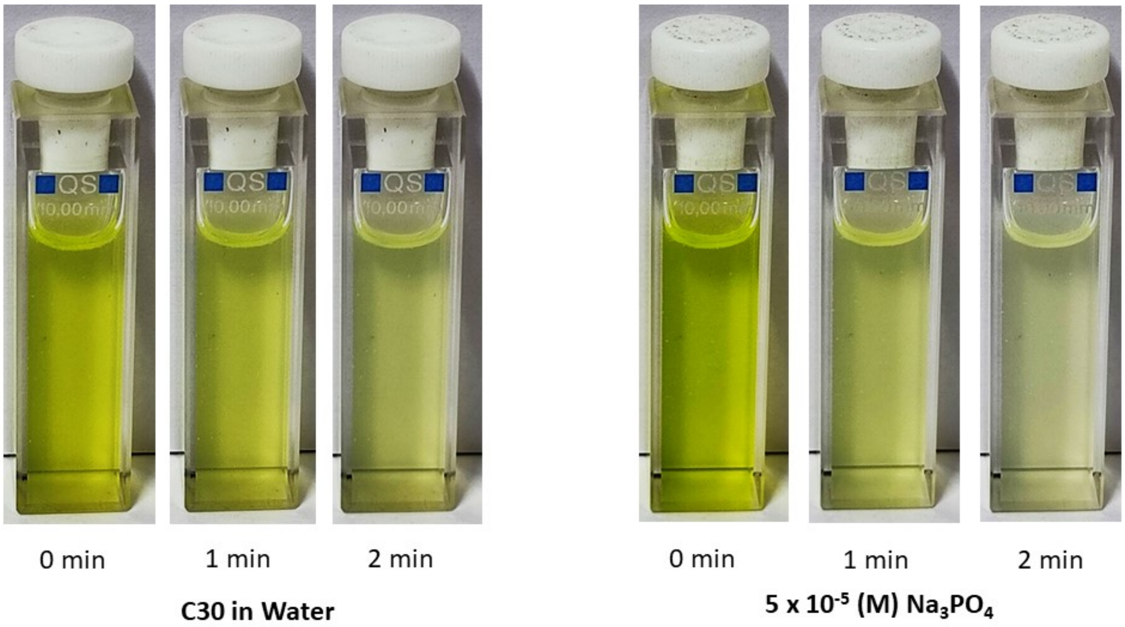

Figure S4: Snap shots of C30 in water and in 5 × 10^{-5} (M) Na_3PO_4 solution at different times.

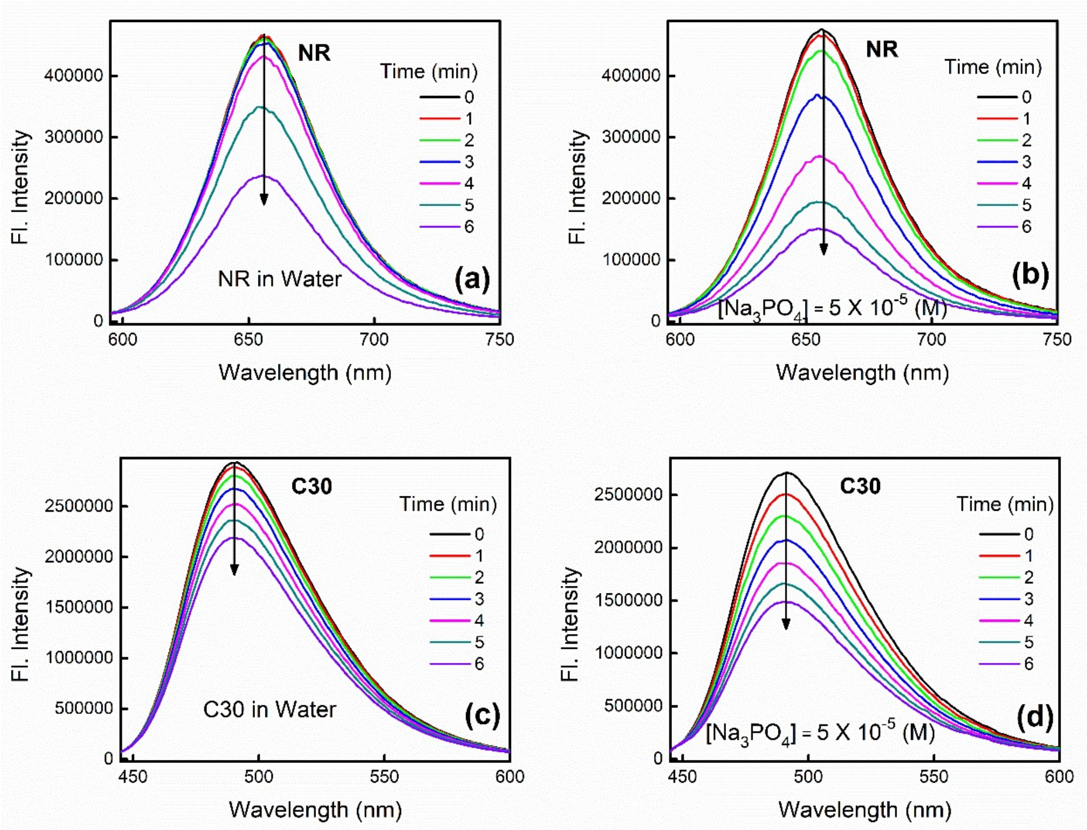

Figure S5. Fluorescence spectra of NR in (a) water and (b) 5 x 10^{-5} (M) Na_3PO_4 solution with increasing time. Fluorescence spectra of C30 in (c) water and (d) 5 x 10^{-5} (M) Na_3PO_4 solution with increasing time.

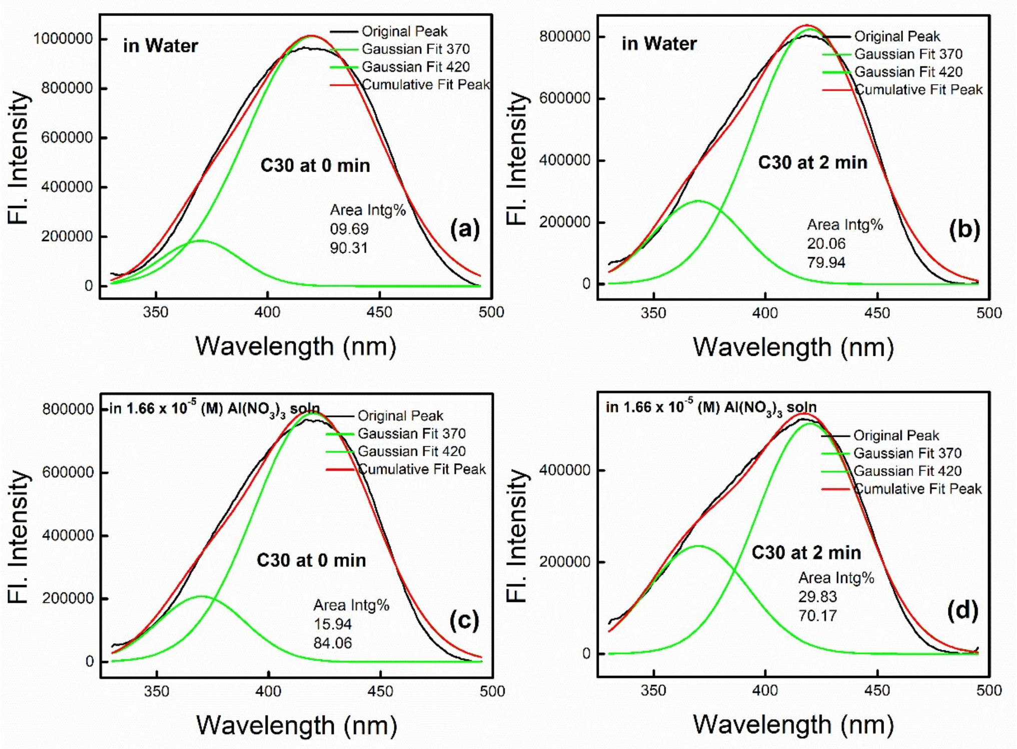

Figure S6. Resolved fluorescence excitation spectra of C30 ((a) and (b)) at λ_{em} = 505 nm in water at different times. Resolved fluorescence excitation spectra of C30 ((c) and (d)) at λ_{em} = 505 nm in 1.66 $\times$ 10^{-5} (M) $Al(NO_3)_3$ solution at different times.

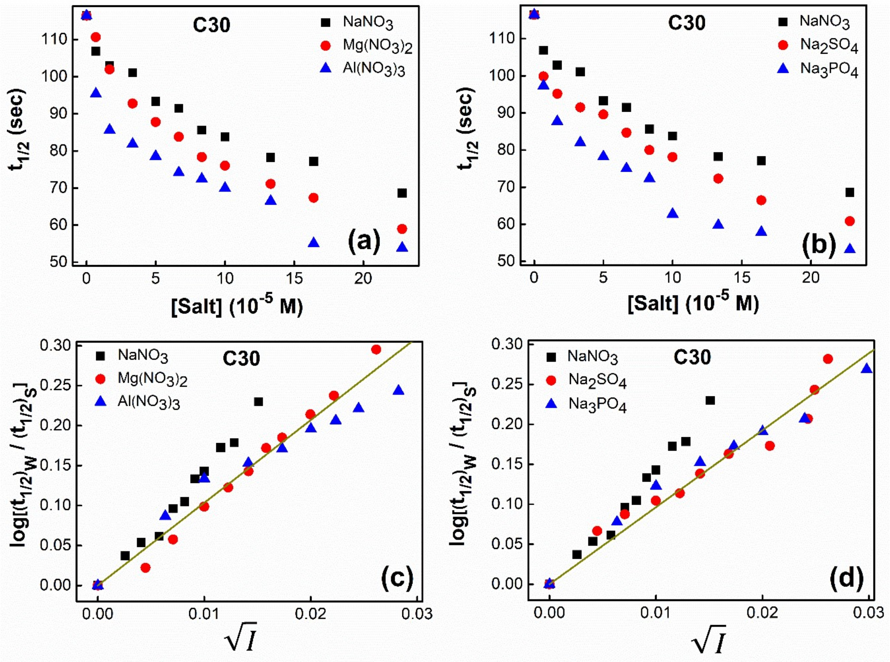

Figure S7. Variation of half-life ($t_{1/2}$) vs. salt concentration for C30 with a variation of (a) cation and (b) anion; (c) and (d) gives plots of log $\left(k/k^0\right)$ vs $\sqrt{I}$ for C30, where $k/k^0 = \left(t_{1/2}\right)_W / \left(t_{1/2}\right)_S$.

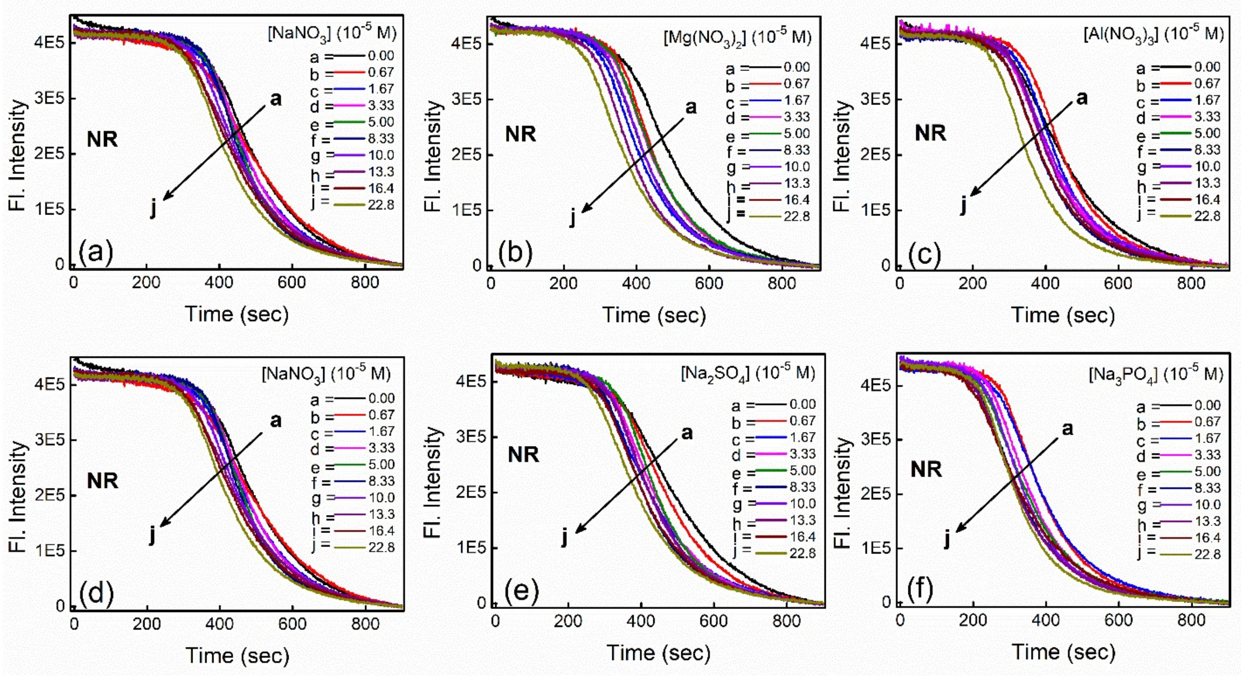

Figure S8. Fluorescence kinetic decay of NR at λ_{em} = 656 nm in water with different salt concentrations (λ_{ex} = 580 nm).

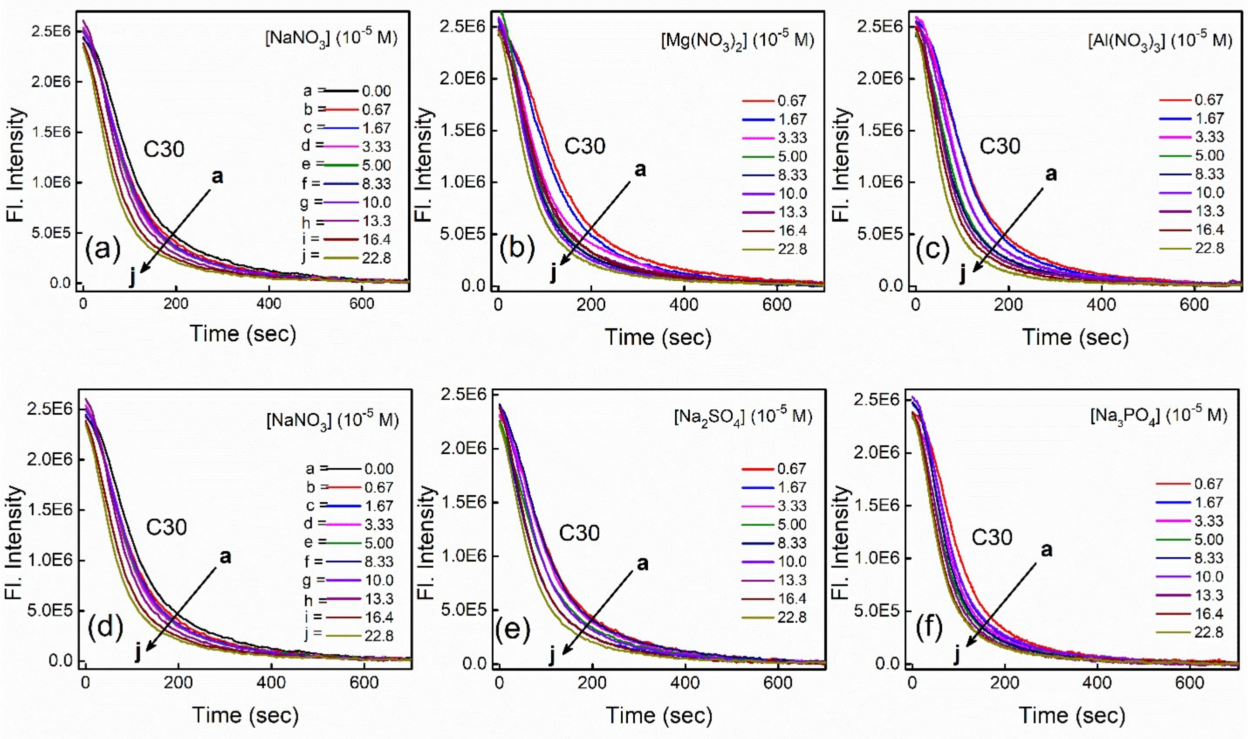

Figure S9. Fluorescence kinetic decay of C30 at λ_{em} = 495 nm in water with different salt concentrations (λ_{ex} = 430 nm).
